# Multimodality treatment on gastric cancer with liver metastasis: case report

**DOI:** 10.3389/fonc.2025.1578314

**Published:** 2025-11-03

**Authors:** Mengnan Li, Gongchen Wang, Lianyu Chen, Liping Zhuang, Hao Chen

**Affiliations:** ^1^ Department of Medical Oncology, Affiliated Hospital of Gansu University of Traditional Chinese Medicine, Lanzhou, China; ^2^ Department of Integrative Oncology, Fudan University Shanghai Cancer Center, Shanghai, China

**Keywords:** immune checkpoint inhibitors, gastric cancer, liver metastasis, cryoablation, case report

## Abstract

**Background:**

Advanced gastric cancer has a devastating prognosis, However, the optimal treatment for gastric cancer patients with multiple liver metastases(GCLM) remains yet to be fully elucidated. Cryoablation is a novel therapeutic approach that has the potential to induce tumor necrosis and elicit anti-tumor immune responses.

**Case presentation:**

A 44-year-old male was diagnosed with advanced gastric adenocarcinoma. Despite undergoing multiple lines of systemic therapy and repeated locoregional treatments, the patient exhibited rapid tumor progression. Subsequent treatment with PD-1 inhibitors demonstrated limited efficacy. However, following the innovative application of cryoablation to eradicate a more significant lesion within the liver metastases, the patient achieved a partial response and experienced 15 months of progression-free survival (PFS) with the combined use of PD-1 inhibitors and chemotherapy at the time of this report.

**Conclusion:**

Cryoablation combined with immunotherapy may provide potential benefits for specific patients and offer a meaningful therapeutic strategy for patients with advanced gastric cancer and liver metastases, which is worthy of further research.

## Introduction

Gastric carcinoma (GC) ranks as the fifth most prevalent malignant tumor and is the fifth leading cause of cancer-related mortality worldwide (1).In China, It is estimated 260,400 deaths in 2022, accounting for nearly half of the global total deaths from GC (2). The prognosis for GC is generally poor, as the majority of patients are diagnosed at advanced stages, often with invasive or metastatic disease. In this context, palliative chemotherapy is highly recommended, and multi-line sequential chemotherapy is considered the standard guideline therapy for patients with unresectable GC (3). The groundbreaking discovery of immune checkpoints has revolutionized the field of cancer therapeutics. In 2023, the Chinese Society of Clinical Oncology (CSCO) guidelines recommended PD-1 inhibitors as the first-line treatment for locally advanced and/or metastatic GC, based on the encouraging results from recent clinical evidence and the outcomes of phase III trials (4–6). Notably, the therapeutic efficacy of PD-1 inhibitors is often constrained by the immunosuppressive tumor microenvironment, posing a significant challenge for GC patients. Consequently, ongoing research is focused on exploring combination strategies that integrate PD-1 inhibitors with other anti-tumor therapies to enhance treatment efficacy.

At present, the optimal therapeutic strategy for gastric cancer patients with liver metastases(GCLM) remains controversial. Multimodality treatment including aggressive first-line chemotherapy with the triplet regimen have emerged as the predominant therapeutic strategies. Several minimally invasive techniques have been proposed for the management of hepatic metastases, such as transarterial chemoembolization (TACE), energy-based ablation (radiofrequency, microwave or cryoablation), radiotherapy, etc. (7). Among these, cryoablation demonstrates distinct advantages, including the capacity to produce larger and more precise ablation zones, a more clearly identifiable therapeutic area, and the potential to stimulate systemic antitumor immune responses through the induction of ectopic tumor-suppressive effects. While the efficacy of cryoablation has been well-documented in malignancies such as liver cancer, lung cancer, and prostate cancer (8), its application in GCLM remains largely unexplored.

This case report presents a patient with advanced GC who exhibited disease progression despite multi-line systemic therapy and repeated locoregional therapy. The patient received anti-programmed cell death-1 (PD-1) antibody, which demonstrated limited efficacy. After the innovative application of cryoablation targeting a more significant lesion within the liver metastasis, the patient achieved a favorable response when treated with a combination of PD-1 inhibitors and chemotherapy.

## Case description

In July 2022, a 44-year-old male patient presented with persistent upper abdominal pain. The patient denied any family history of cancer and had no history of hypertension, diabetes, or hepatitis. A computed tomography (CT) scan identified a malignant subserosal tumor on the greater curvature of the stomach, accompanied by multiple enlarged peri-gastric lymph nodes and multiple liver metastases. A painless gastroscopy revealed an ulcerative lesion on the greater curvature of the stomach. Histopathological examination of the gastric biopsy specimens confirmed the presence of poorly differentiated adenocarcinoma of the stomach.

Immunohistochemical analysis of the gastroscopic biopsy specimens demonstrated the following markers: pMMR, HER2(-), Ki60(90%+), CK7(+), CK20(+), SATB(+), and CDX2(+). Based on these comprehensive diagnostic results, the patient was clinically diagnosed with stage IV gastric adenocarcinoma with multiple liver metastases, according to AJCC 8(th) edition TNM staging system (9).

Subsequently, the patient commenced systemic chemotherapy with the oxaliplatin and S−1(SOX) as the first−line treatment. According to the response evaluation criteria in solid tumors (RECIST 1.1), the patient was evaluated as radiologic stable disease (SD) after the 2nd, 4th, and 6th cycles of treatment respectively. However, after the 7th course of chemotherapy, his serum AFP level surged to exceeding 3630 ng/ml. A follow-up contrast-enhanced CT scan revealed that the tumor extended from the subserosal layer to beyond the serosal layer, and new metastatic lesions in the liver. Therefore, the clinical response was assessed as disease progression according to the RECIST 1.1 guidelines.

According to the CSCO guidelines for advanced GC, patients with liver metastasis may benefit from local treatment. In order to target the live lesions, microwave ablation and TACE were performed sequentially in the case of adequate liver function reservation on February 9, 2023. After these minimally invasive procedures, the chemotherapy regimen was adjusted to Paclitaxel (300mg day 1) plus S-1 (60 mg bid days 1–14) (TS). Over the subsequent two months, the patient completed two cycles of TS chemotherapy without serious adverse reactions. However, a follow-up enhanced magnetic resonance imaging (MRI) scan on May 5 revealed that the liver lesions remained active. Consequently, the treatment regimen was modified to FLOT(fluorouracil plus leucovorin, oxaliplatin and docetaxel, q2w) in combination with PD-1 inhibitor (sintilimab 200mg day 1). The primary side effects included grade III myelosuppression in combination with fever after two cycles of intensive chemotherapy. Additionally, the levels of CEA and CA199 tumor markers showed a gradually increasing trend throughout the treatment course. During the third cycle of FLOT chemotherapy, the patient experienced flushing, pruritus, and abdominal pain after 15 minutes of starting the oxaliplatin infusion. Oxaliplatin was subsequently discontinued, and the patient was administered 5-FU without further complications.

Given the immunomodulatory effects of cryoablation and the potential synergistic anti-tumor effects between cryoablation and immunotherapy, we hypothesized that this combination could offer significant therapeutic benefits. On June 13, 2023, we performed ultrasound-guided cryoablation of one 3cm large metastatic tumor in the right lobe of the liver(the other lesions and lymph nodes were not subjected to cryoablation) using a nitrogen-ethanol-based Co-ablation system. Complete ablation was achieved after double freeze-thaw cycles(15 min freeze, 5 min thaw) under intravenous anesthesia, and the patient was discharged in stable condition without significant complications. The PD-1 inhibitor therapy (sintilimab) was continued as scheduled. In light of the patient’s prior hypersensitivity reactions to oxaliplatin, the systemic chemotherapy was adjusted to intravenous docetaxel, and hepatic artery infusion chemotherapy(HAIC) with infusional leucovorin and fluorouracil. This modified treatment plan was administered without any adverse events, demonstrating favorable tolerability and safety.

**Figure 1:**
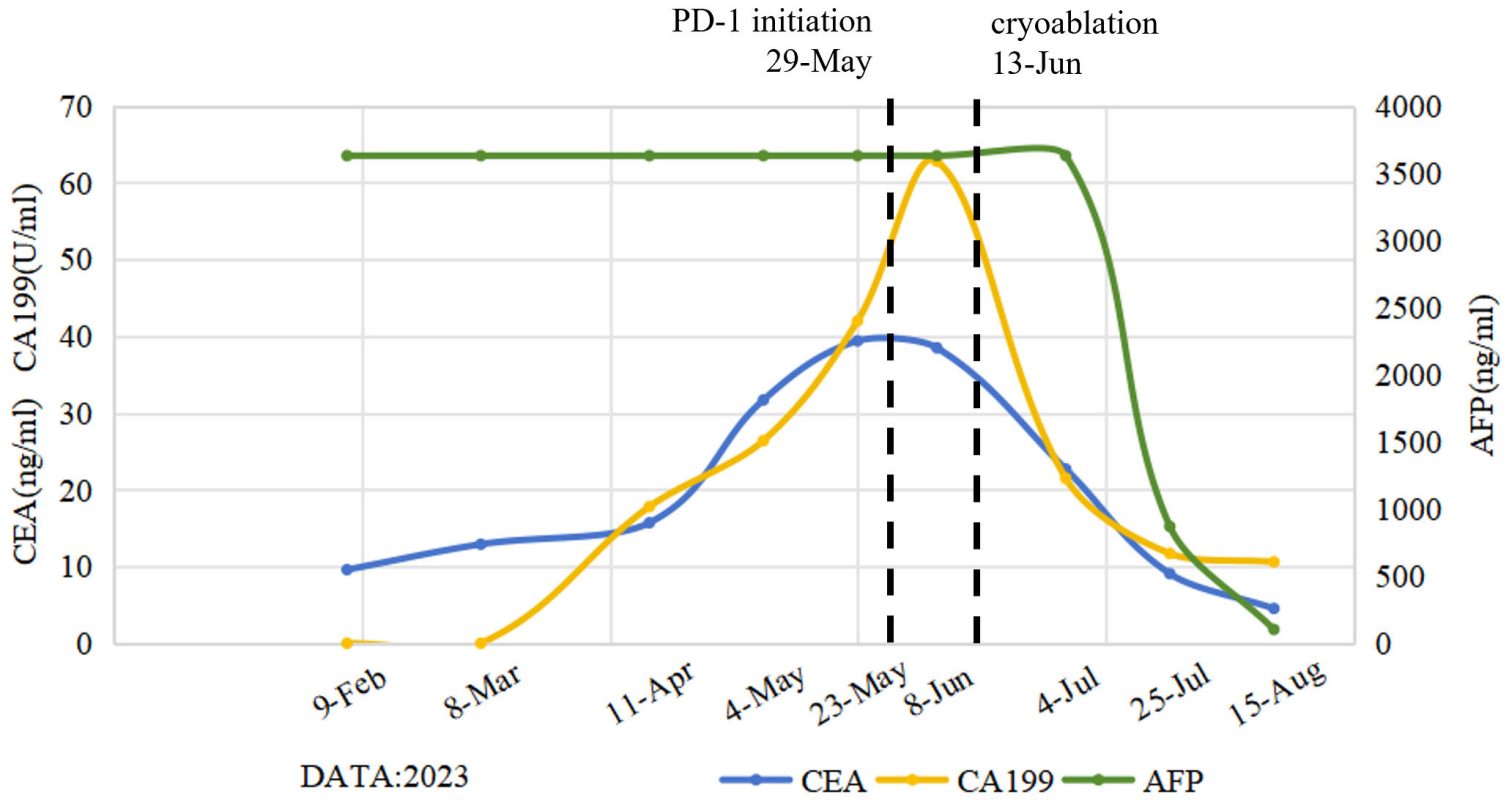
Dynamic change curves of peripheral blood tumor marker levels following the initiation of multi-line treatment. The levels of CEA and CA199 tumor markers showed a gradually increasing trend during the previous treatment regimens, peaking at the time of failure of the initial immune checkpoint inhibitor (ICI) therapy. Subsequently, these levels showed a gradually decreasing trend after cryoablation. Notably, AFP levels revealed a significant and sustained reduction throughout the post-cryoablation treatment phase. CEA, carcinoembryonic antigen; CA199, Carbohydrate antigen 19-9; AFP, alpha-fetoprotein.

**Figure 2:**
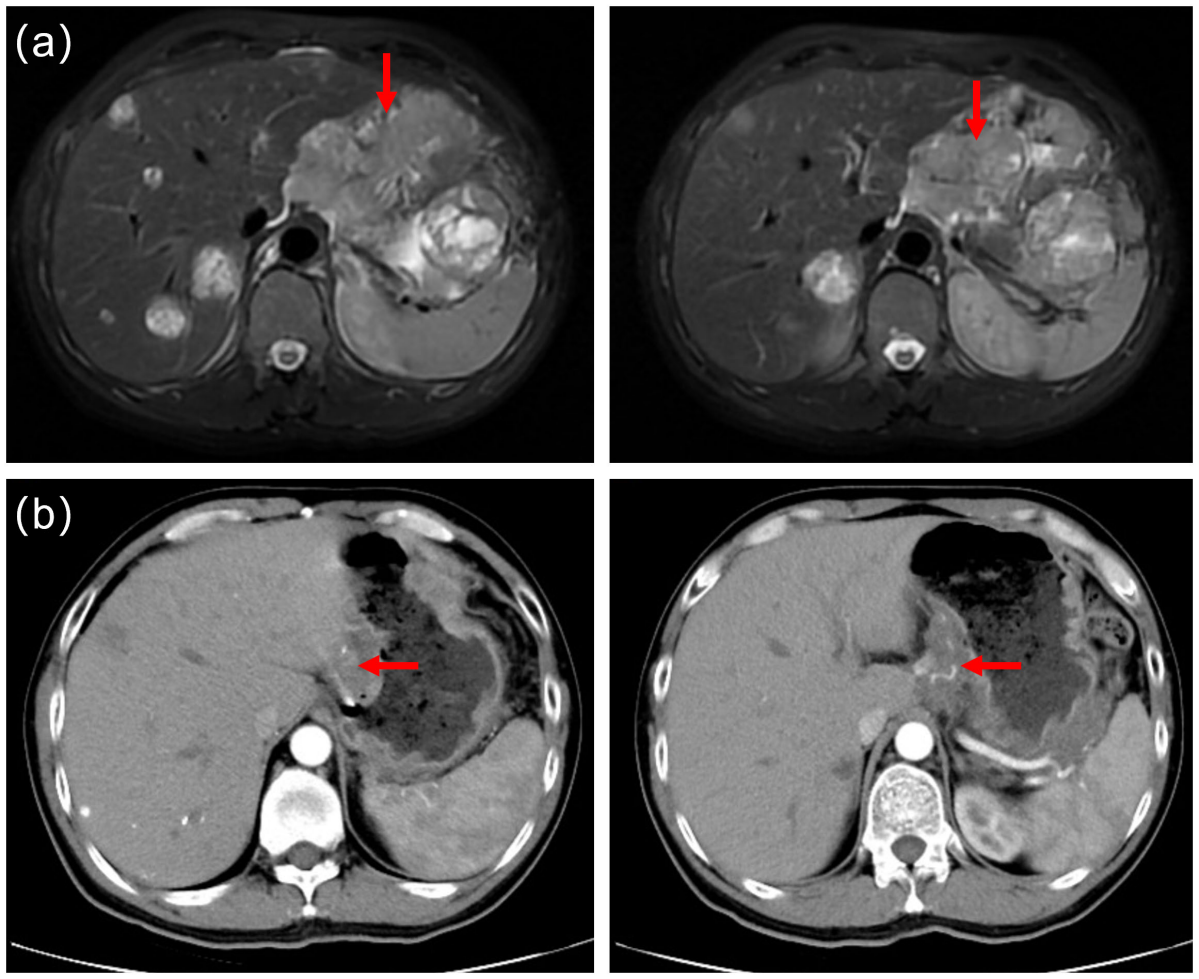
**(a)** Prior to cryoablation, abdominal magnetic resonance imaging(MRI) identified multiple liver metastases and metastatic lymph nodes. **(b)** Follow-up computed tomography scans in August 2023 revealed a significantly reduced in liver tumor lesions. The shortest diameter of the largest hepatogastric space lymph nodes shrunk from 46mm to 24mm(reduction by 47.83%).

Routine follow-up examinations were conducted after two cycles of treatment, revealing a marked improvement in the patient’s condition. The serum AFP level markedly decreased, plummeting from >3630ng/ml to 103ng/ml (**Figure 1**). In August 2023, abdominal CT imaging further confirmed a substantial reduction in the liver lesion size, with the shortest diameter of the largest hepatogastric space lymph nodes shrinking from 46mm to 24mm(reduction by 47.83%) (**Figure 2**). The clinical outcome was classified as partial remission per the RECIST 1.1 guidelines.

**Figure 3:**
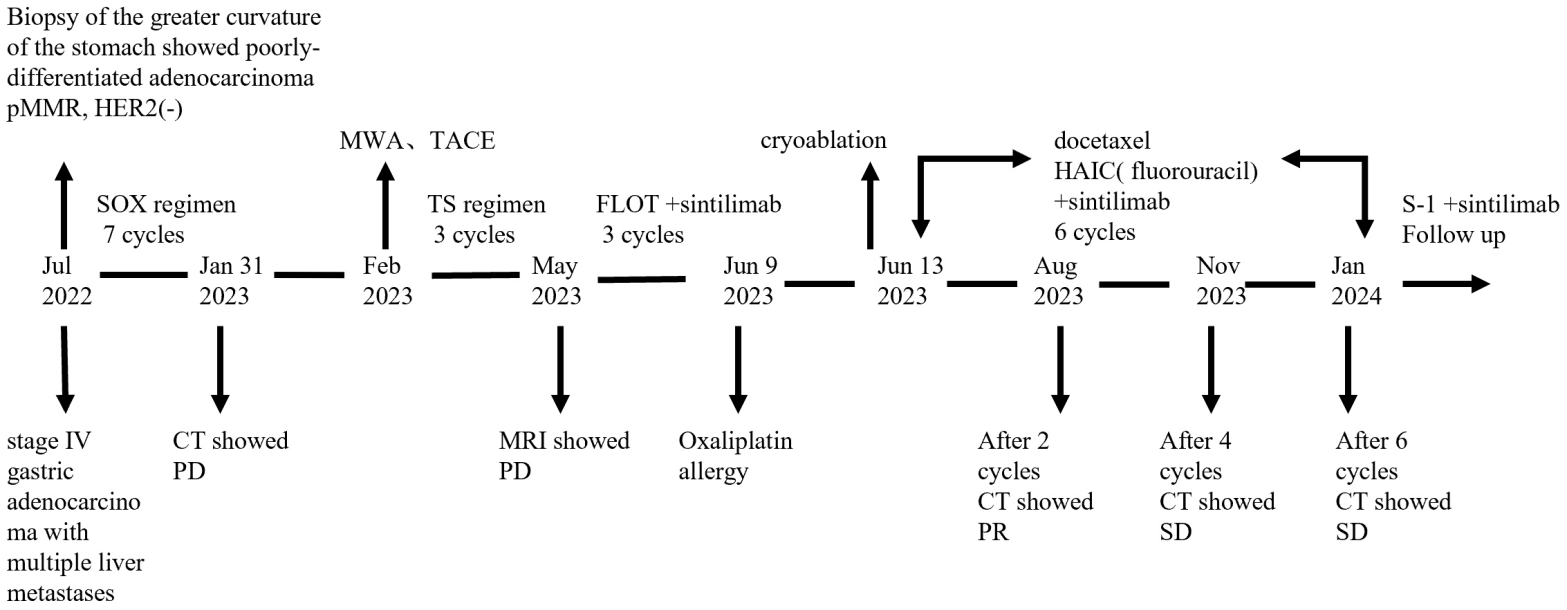
Timeline figure of the clinical course.

The patient continued to receive chemotherapy and immune checkpoint inhibitor therapy, along with regular follow−up evaluations. Enhanced CT scans performed in November 2023 and January 2024 demonstrated stable lesions, with no significant changes. Laboratory tests showed normal thyroid, hepatic and renal function, and the patient did not experience any grade III-IV myelosuppression or gastrointestinal reactions. After six cycles of the above therapy, the patient transitioned to maintenance therapy of S-1(60mg bid, days 1-14) and Sintilimab(200 mg, d1) every 3 weeks to ensure sustain remission. During this treatment period, the patient experienced no significant discomfort or adverse events related to chemotherapy and immunotherapy. At the time of this report, the patient had achieved a progression-free survival (PFS) of 15 months. A detailed timeline figure of the clinical course is shown in **Figure 3**.

## Discussion

GCLM is a common and highly challenging form of advanced gastric cancer, which remains a major cause of death related to GC. Palliative systemic therapy plays an important role in patients with GCLM, and it includes chemotherapy, immunotherapy, targeted therapy. With the advancement of multidisciplinary team (MDT) management, local treatments such as palliative surgery, radiotherapy, energy-based ablation, intraperitoneal perfusion, and arterial interventional embolization perfusion may further contribute to prolonging survival and improving quality of life.

Cryoablation is recognized as a safe and effective palliative treatment for liver metastasis. A retrospective cohort study involving 19 patients who underwent cryoablation for liver metastases following gastrectomy for gastric cancer revealed a median overall survival(OS) of 16 months and a median local tumor PFS of 8 months. Additionally, The quality of life for patients was improved after cryoablation (*P* < 0.05). It can provide good local control and improve the quality of life for patients (10). Preclinical data suggested that cryoablation may have a better immunogenic potential than radiofrequency ablation or microwave ablation, and induce the most significant increase in the percentage of CD8+T cells (11). In recent years, preclinical studies offer preliminary insights into the potential of cryoablation combined with immunotherapy in cancer treatment (12, 13). A retrospective review suggests that this combined therapeutic approach can be feasible and safe for both palliative and local control according to the indications (14). Although a series of related clinical trials are currently underway, we haven’t seen clinical application of cryotherapy with adjuvant PD-1 in GCLM. This report presents a case of GCLM in which partial remission was achieved through a novel combination of cryoablation and immune checkpoint inhibitors. Interestingly, the patient achieved sustained clinical benefits following treatment with immune checkpoint inhibitors and chemotherapy, which persisted up to the time of this report. At the time of this report, the patient had achieved a PFS of 15 months, highlighting the potential of this combined therapeutic approach.

In this report, cryoablation destroys tumor tissue through a process of rapid freezing and thawing using Co-ablation system. The repeated freeze-thaw cycle of cryoablation stimulates the release of antigens and cytokines, such as interferon (IFN)-γ, TNF-α, IL-2 and IL-12, which are then taken to dendritic cells, leading to further acquired immune activation (15). A retrospective study compared the efficacy of cryoablation alone and cryoablation combined with nivolumab (PD-1 antibody) in the treatment of advanced NSCLC. Patients in cryo-nivolumab group had a significant improvement in immune function and short-term efficacy (*P* < 0.05). The levels of CTCs and tumor markers CYFRA21–1 and NSE in cryo-nivolumab group were reduced significantly (*P* < 0.05). This provides strong clinical evidence for the synergistic effect of cryoablation and ICIs (16). In this case, AFP levels revealed a significant and sustained reduction throughout the post-cryoablation treatment phase. Unfortunately, pre-and post-treatment immune cell analysis (e.g., CD8+/CD4+ ratio, dynamic changes in PD-L1 expression) was not obtained due to the limitations of clinical laboratory conditions. Therefore, prospective studies with larger sample sizes on patients with GCLM will be necessary in the future to validate this combination strategy. Cryoablation offers the unique advantage of preserving the immunogenicity of tumor antigens, which can be recognized by the host immune system to induce the greater immune response, thereby causing the abscopal effects (17, 18). This case demonstrates that cryoablation can cause an abscopal effect, evidenced by the observed response in peri-gastric lymph nodes that were not directly subjected to cryoablation.

Considering the immunomodulatory effects of cryoablation, numerous studies have explored its potential synergistic effects with immunotherapy. Clinical and experimental studies have also found that cryoablation may cause immune suppression or unresponsiveness, which is strongly related to cancer type, the ratio of necrosis/apoptosis, and cryoablation parameters (19). However, there are no recommend criteria regarding the selection of drug dosage, the sequence of drug administration, or the timing of cryoablation, all of which warrant further research and exploration. Furthermore, the immune response elicited is generally non-durable and often inadequate to eradicate distant metastatic lesions. Therefore, It is necessary to continuously optimize and explore combined treatment strategies to enhance the clinical efficacy of immune checkpoint inhibitors (ICIs).

HAIC and TACE had emerged as a promising therapeutic approach for liver metastases, offering the advantage of delivering high local drug concentrations to the tumor site while minimizing systemic side effects. While some studies have demonstrated its clinical efficacy, the routine application of HAIC for the management of liver metastases necessitates further validation through large-scale prospective clinical trials (20). HAIC may be a viable option for patients with GCLM who are unable to tolerate more intensive systemic chemotherapy regimens. This case suggests that HAIC could be a reasonable option for patients with GCLM, even in extensive intrahepatic metastasis of treatment. The potential efficacy of HAIC recipients should be evaluated through multidisciplinary discussion, giving full play to technical advantages to strive for a longer survival and better quality of life.

In summary, this patient underwent locoregional and systemic therapies, achieving a significant clinical response through multimodality therapy. With the development of interventional oncology techniques, immunotherapy-based combination regimens provide a meaningful therapeutic strategy. However, it is important to acknowledge that such combination therapies are associated with substantial financial costs, with its efficacy varies from person to person. Consequently, further clinical studies are need to discover the biomarker for clinical outcome prediction based on the multimodal treatments and to establish standardized protocols for their application.

## Data Availability

The original contributions presented in the study are included in the article/supplementary material. Further inquiries can be directed to the corresponding author.
