# Supplementary figures and images for: Multimodality treatment on gastric cancer with liver metastasis: case report
Source: Front Oncol. 2025 Nov 3;15:1578314. doi: 10.3389/fonc.2025.1578314 (PMC12620203; doi:10.3389/fonc.2025.1578314)

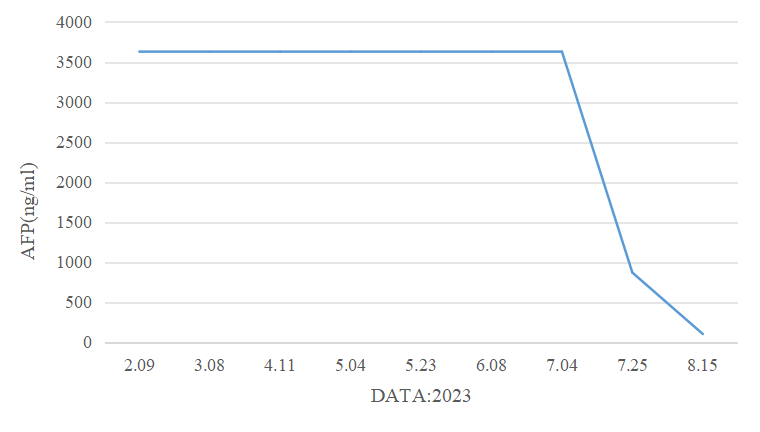


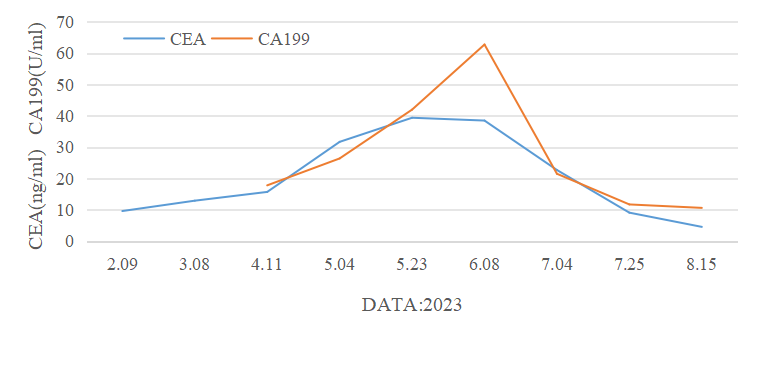

Supplement: Supplementary file 1 [file DataSheet1.doc]

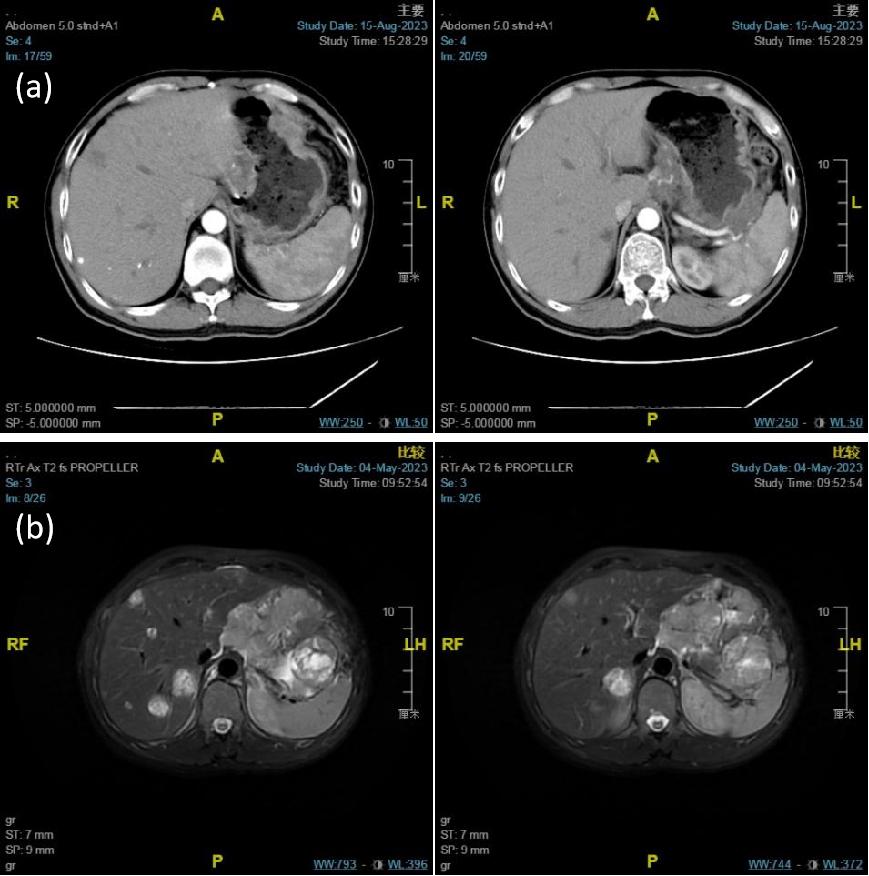

Supplement: Supplementary file 2 [file Image1.jpeg]
